# The Presynaptic Microtubule Cytoskeleton in Physiological and Pathological Conditions: Lessons from *Drosophila* Fragile X Syndrome and Hereditary Spastic Paraplegias

**DOI:** 10.3389/fnmol.2016.00060

**Published:** 2016-07-25

**Authors:** Felipe J. Bodaleo, Christian Gonzalez-Billault

**Affiliations:** ^1^Laboratory of Cell and Neuronal Dynamics, Department of Biology, Faculty of Sciences, Universidad de ChileSantiago, Chile; ^2^Center for Geroscience, Brain Health and Metabolism (GERO)Santiago, Chile; ^3^The Buck Institute for Research on Aging, NovatoCA, USA

**Keywords:** microtubules, presynaptic terminals, neurotransmitter release, active zone, *Drosophila’s* neuro muscular junction

## Abstract

The capacity of the nervous system to generate neuronal networks relies on the establishment and maintenance of synaptic contacts. Synapses are composed of functionally different presynaptic and postsynaptic compartments. An appropriate synaptic architecture is required to provide the structural basis that supports synaptic transmission, a process involving changes in cytoskeletal dynamics. Actin microfilaments are the main cytoskeletal components present at both presynaptic and postsynaptic terminals in glutamatergic synapses. However, in the last few years it has been demonstrated that microtubules (MTs) transiently invade dendritic spines, promoting their maturation. Nevertheless, the presence and functions of MTs at the presynaptic site are still a matter of debate. Early electron microscopy (EM) studies revealed that MTs are present in the presynaptic terminals of the central nervous system (CNS) where they interact with synaptic vesicles (SVs) and reach the active zone. These observations have been reproduced by several EM protocols; however, there is empirical heterogeneity in detecting presynaptic MTs, since they appear to be both labile and unstable. Moreover, increasing evidence derived from studies in the fruit fly neuromuscular junction proposes different roles for MTs in regulating presynaptic function in physiological and pathological conditions. In this review, we summarize the main findings that support the presence and roles of MTs at presynaptic terminals, integrating descriptive and biochemical analyses, and studies performed in invertebrate genetic models.

## Introduction

Every motor, cognitive, and association function executed by the nervous system relies on the establishment of neuronal networks that involve the generation, maintenance, and pruning of synaptic contacts. The animal nervous system possesses a diverse array of synapses characterized by their structure and chemical nature, but in general terms, a synaptic contact is defined as a junction between a presynaptic neuron and a postsynaptic cell, that can be another neuron, a muscle cell or a gland cell (Jessell and Kandel, 1993). At the subcellular level, synapses are composed of an axonal presynaptic terminal, that releases neurotransmitters in response to an action potential, and a postsynaptic terminal which receives and integrates the synaptic input (Garner et al., 2000; Südhof, 2012). The highly specialized structure and morphology of pre- and post-synaptic assembly depends on several factors, including cytoskeleton dynamics. Actin microfilaments are the major cytoskeletal component of glutamatergic synapses in mature neurons. In presynaptic terminals, actin regulates synaptic vesicle (SV) pool dynamics, including their mobilization to the active zone, endocytosis, and exocytosis, as well as providing a scaffolding system for the spatial organization of regulatory elements in the nerve terminal (Colicos et al., 2001; Shupliakov et al., 2002; Sankaranarayanan et al., 2003). The actin cytoskeleton is also highly enriched in postsynaptic dendritic spines, where it regulates spinogenesis and the structural plasticity observed in mature neurons in response to activity (Matus et al., 1982; Matus, 2000; Yuste and Bonhoeffer, 2004). In contrast, the presence and function of the synaptic microtubule (MT) cytoskeleton is barely beginning to be understood. During the last decade, the transient invasion of MTs to a small percentage of dendritic spines in mature neurons in response to synaptic activity has been demonstrated (Hu et al., 2008; Jaworski et al., 2009). MTs entry into dendritic spines promotes their stabilization through the recruitment of the postsynaptic protein-95 (PSD-95; Hu et al., 2011) and the activation of NMDA glutamate receptors (Merriam et al., 2011). These findings set a turning point from the widespread notion that MTs are absent from dendritic spines, a notion based on the technical difficulties of detecting MTs at postsynaptic terminals by electron microscopy (EM; Gray et al., 1982; Landis and Reese, 1983; Fiala et al., 2003), despite the fact that MT had been detected in dendritic shaft by several different techniques (Caceres et al., 1983; Spacek and Harris, 1997; Kaech et al., 2001). However, the presence and function of MTs at presynaptic terminals are beginning to be understood, supported by evidence derived from studies performed mainly in vertebrate central synapses and in neuromuscular junctions (NMJ) of *Drosophila* larvae. In this review, we will summarize the ultrastructural and biochemical data that endorse the notion of a presynaptic MT cytoskeleton in vertebrate synapses, and the MT-dependent regulation of presynaptic structure and physiology at NMJs in *Drosophila*. Finally, we will address the use of the *Drosophila* NMJ as a model for the study of several neurodegenerative disorders that underlie MT cytoskeleton-related mechanisms in pathological conditions.

## The mt Cytoskeleton Is A Constituent of Presynaptic Terminals

### Descriptive Analyses Revealed the Presence of MTs in Presynaptic Terminals

Presynaptic neuronal MTs were first described by EM techniques as fibrillar structures that were coupled to SVs along axons (Schmitt, 1968). Those first observations relating MT and presynaptic terminals were undertaken in central nervous system (CNS) neurons derived from lamprey larvae (*Petromyzon marinus*). In those samples, MTs were associated with SVs in the proximity of synaptic junctions in axonal terminals, but absent from neurotransmitter release zones. Additionally, no apparent contacts with the presynaptic plasma membrane were found (Smith et al., 1970). In parallel, the first insights into the existence of tubulin (previously referred to as “MT protein”), were biochemically determined in subcellular fractions derived from nerve endings. In those preparations, tubulin accounted for an important fraction of total soluble protein (Feit and Barondes, 1970; Feit, 1971; Lagnado et al., 1971), and was also associated with the presynaptic membrane (Gozes and Littauer, 1979). Work performed over subsequent years could not unequivocally conclude that MTs were present in presynaptic terminals, very likely due to technical issues derived from the use of conventional protocols based on glutaraldehyde fixation and osmium tetroxide post-fixation (Gray and Willis, 1970; Scott and Guillery, 1974). Interestingly, the development of a novel sample preparation protocol, where tissue was incubated with an albumin solution prior to fixation, enabled the detection of MTs in presynaptic fractions associated with both SVs and dense core vesicles (DCVs), which were in close apposition to the presynaptic plasma membrane, and apparently reaching the active zone (Gray, 1975; Bird, 1976). These surprising findings were corroborated some years later by using a rapid tissue freezing and acetone substitution fixation protocol (Hirokawa and Kirino, 1980) or a cold pre-fixation treatment (Jones et al., 1980). These presynaptic MTs are characterized by being spaced closer together than the MTs present in axons, and they seem to expand to form vesicle-filled varicosities in the immediate presynaptic region (Bartlett and Banker, 1984). The fact that presynaptic MTs are labile and that they require specific protocols for visualization is related with their dynamic properties. In fact, fixation procedures performed with calcium chelators (e.g., EDTA or EGTA) preserved the structure of presynaptic MTs (Chan and Bunt, 1978; Gordon-Weeks et al., 1982), which is closely related with the fact that calcium ions destabilize MTs (Schlaepfer, 1971; Weisenberg, 1972; O’Brien et al., 1997). Despite the weight of ultrastructural and biochemical evidence that suggest the presence of MTs in presynaptic terminals, immunohistochemical analyses revealed that both MTs and high molecular weight MAPs are absent from nerve terminals in the mammal brain (Matus et al., 1975, 1981), even though MTs are detected in presynaptic synaptosomes by immunofluorescence (Cumming et al., 1983). This is a striking observation since first attempts to determine the presence of MTs in synaptosomal preparations through EM failed using cold fixation techniques (Blitz and Fine, 1974; Kadota et al., 1976). Nevertheless, other groups described MTs interacting with a subset of horseshoe-shaped mitochondria in synaptosomes, forming a fibrillar ring around these organelles (Chan and Bunt, 1978; Hajos et al., 1979). These contrasting descriptions could be explained by the fact that synaptosomal MTs seem to destabilize when exposed to cold temperatures, and to stabilize when fixation protocols are performed at room temperature (Hajos et al., 1979). A similar observation was found in presynaptic terminal preparations derived from mammal Calyx of Held synapses by electron tomography, where MTs form a mitochondrion-associated adherens complex (MAC) that determines the precise anchoring of these organelles in the presynaptic terminal (Perkins et al., 2010). By immunocytochemistry, prominent MT bundles tracking with neurofilaments at the Calyx presynapse can be observed (Paysan et al., 2000). In mammalian ribbon synapses from retina photoreceptors and bipolar cells, the presence of the component of motor kinesin II, KIF3A, that is expressed at the presynaptic ribbon and associated SVs has been determined, suggesting the presence of MT rails (Muresan et al., 1999). However, in classic EM approaches, MTs are only detectable traversing eccentrically the *en passant* presynaptic terminals at retinal bipolar cells (Strettoi et al., 1990). It is worth mentioning that in goldfish bipolar retinal cells, a thick band of MTs emerges from the axon and loops throughout the presynaptic terminal where it interacts with mitochondria, a mechanism that may be involved in mitochondria positioning in order to provide an energy supply for continuous neurotransmitter release (Graffe et al., 2015).

### Interaction of Tubulin with Presynaptic Components

The biochemical evidence that related MTs with presynaptic functions became apparent, since tubulin directly interacts with the presynaptic protein Synapsin I (Nakayama and Silverman, 1986). Synapsin proteins form a family of neuronal phosphoproteins that cluster SVs to cytoskeletal elements at presynaptic terminals, regulating SV cycling and neurotransmitter release (Greengard et al., 1993; Ryan et al., 1996). Synapsin I contains two domains for interacting with tubulin, which are located in the globular head amino-terminal region of the protein and in the elongated tail domain (Bennett et al., 1991; Petrucci and Morrow, 1991). It has been demonstrated that Synapsin interacts with the actin cytoskeleton in a highly regulated manner, where depolarization induces calcium-dependent phosphorylation of Synapsin by CaMKII in residues S566 and S603, which ultimately results in a dramatic decrease in Synapsin–actin interaction (Huttner and Greengard, 1979; Bähler et al., 1989; Cesca et al., 2010). However, phosphorylation of Synapsin in S23 mediated by either mitogen-activated protein (MAP) kinase and/or Cyclin-dependent kinase 5 (Cdk5), does not affect the Synapsin MT bundling activity or the interaction with MTs (Matsubara et al., 1996). Importantly, it is likely that phosphorylation in as yet undiscovered sites, or other post-translational modifications of Synapsin regulate its interaction with the MT cytoskeleton. Further experiments need to be performed to confirm these assumptions. At a structural level, rapidly frozen tissue analyzed by EM revealed for the first time that MT fibers interact with SVs, mitochondria and with other MT filaments in presynaptic terminals through thin strands (Schnapp and Reese, 1982). Years later, these strands were proposed to correspond to single molecules of Synapsin, conforming a molecular link between SVs and MTs at presynaptic terminals (Hirokawa et al., 1989; Gotow et al., 1991).

Another level of MT regulation at the synaptic terminal could be tubulin phosphorylation. Several protein kinases phosphorylate tubulin, such as cyclin-AMP dependent kinases, casein kinases, and tyrosine kinases (MacRae, 1997). Interestingly, it has been shown that calcium–calmodulin kinase (CamKII) is a protein kinase that interacts with SVs in presynaptic terminals (Benfenati et al., 1996; Ninan and Arancio, 2004), and directly phosphorylates both α-tubulin and β-tubulin in enriched synaptosomal fractions (Burke and DeLorenzo, 1981). Such phosphorylation is stimulated by activity (Burke and DeLorenzo, 1982), suggesting a functional link between neurotransmitter release and tubulin post-translational modifications. Also MTs directly bind with the SV membrane protein Synaptotagmin I, where the interaction occurs through the C-terminal region of β-tubulin and both cytoplasmic domains (C2A and C2B) of Synaptotagmin I, which could provide a novel mechanism for attaching SVs to presynaptic MTs (Honda et al., 2002). Although previous work suggests a functional coupling between MTs and neurotransmitter release (Burgoyne and Cumming, 1983; Ashton and Dolly, 1997), further studies are required to resolve this issue.

**FIGURE 1:**
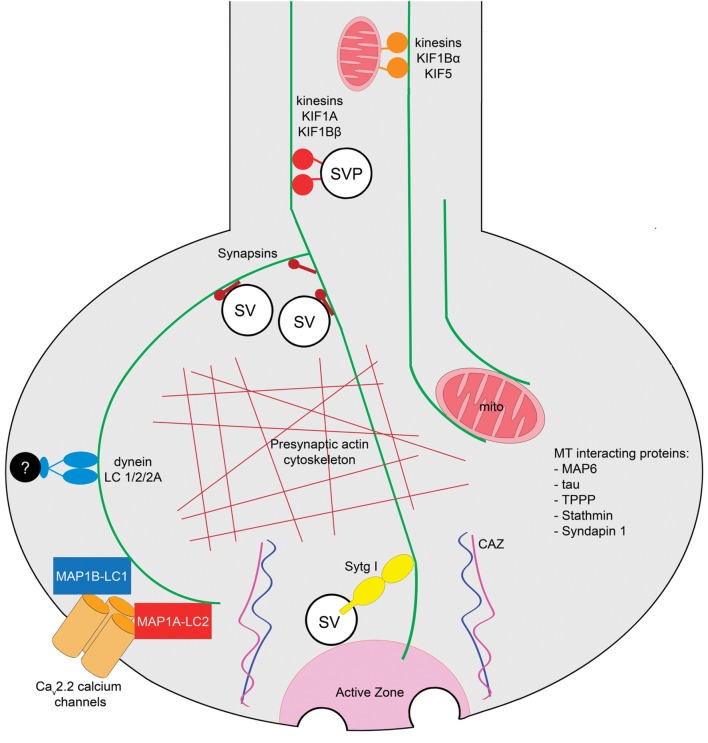
**Roles of MTs at presynaptic terminals of the mammalian central nervous system.** Axonal MT tracks regulate the transport of SVPs and mitochondria to the presynaptic terminals mediated by anterograde motors, kinesins KIF1A and KIF1Bβ, and KIF1Bα and KIF5, respectively. MTs interact and locate mitochondria at presynaptic terminals [EM, electron tomography]. The MT cytoskeleton interacts with Synapsin and with the actin cytoskeleton at presynaptic terminals [co-sedimentation and EM approaches]. Since MTs have been observed in the proximity of active zones, and they interact with the calcium sensor Sytg I, a MT-dependent mechanism in neurotransmission is plausible [pull-down assay]. The light chains of the MT-interacting proteins MAP1B (LC1) and MAP1A (LC2) are present at presynaptic terminals where they interact with the Ca_v_2.2 calcium channel [yeast two hybrid, IF, pulldown, immunoprecipitation approaches]. Additionally, the light chain of the retrograde motor dynein is presynaptically located [mass spectroscopy]. The MT-interacting proteins TPPP, Stathmin, Tau, MAP6, and Syndapin have been detected in the murine presynaptic terminal proteome [mass spectroscopy]. Techniques used to determine each observation are shown in brackets. Abbreviations: CAZ, cytomatrix of the active zone; mito, mitochondria; SV, synaptic vesicle; SVP, synaptic vesicle precursor; Sytg I, synaptotagmin I; TPPP, tubulin polymerization-promoting protein.

The architecture of the subcellular domains that support neurotransmitter release in the presynaptic terminals of the CNS was described initially as a series of dense structures which are organized on the cytoplasmic surface of the presynaptic membrane (Gray, 1963). These dense projections form the “presynaptic vesicular grid,” and were defined as hexagonal organized structures that apparently serve as attachment sites for SVs (Akert et al., 1969; Pfenninger et al., 1972). Contemporary work determined that these structures correspond to the cytomatrix of the active zone (CAZ), a meshwork of proteins that spatially and functionally regulate several steps of the SV cycle (Garner et al., 2000; Dresbach et al., 2003; Gundelfinger and Fejtova, 2012). It has been described that the labile MTs present at presynaptic terminals are arranged in a precise geometrical array in contact with presynaptic dense projections conformed by CAZ (Gray, 1983). In addition to MTs, other MT-interacting proteins are associated with presynaptic components. For example, the light chains of the MT-associated protein 1B (LC1-MAP1B) and MT-associated protein 1A (MAP1A) interact with the presynaptic voltage dependent calcium channel Ca_v_2.2 (Leenders et al., 2008; Gandini et al., 2014). Several groups have performed mass spectrometry of presynaptic preparations, and have found that they contain tubulin (Phillips et al., 2001; Zhai and Bellen, 2004; Morciano et al., 2009). It is therefore possible that MTs could directly interact with components of the active zone allowing the correct delivery of SVs at docking sites or cooperating with the establishment and spatial organization of the CAZ (Laßek et al., 2014). Other studies, using isobaric tags for relative and absolute quantification (iTRAQ) and mass spectroscopy determined that different isoforms of tubulin were present in both free and docked SV fractions of murine synaptosomes, and also verified that the presence of the presynaptic MT cytoskeleton does not depend on the excitatory or inhibitory identity of synapses (Boyken et al., 2013). In addition to tubulin, several proteins that interact with the MT cytoskeleton have also been detected in the presynaptic active zone proteome, such as the MT-reorganizing protein syndapin1, the tubulin polymerization-promoting protein (TPPP), stathmin, the MT-stabilizing proteins tau and MAP-6, and dynein light chain subunits 1/2/2A (Weingarten et al., 2014). The presynaptic MT tracks are also components of a three-dimensional model of an “average” presynaptic terminal (Wilhelm et al., 2014). Interestingly, in NMJs of *Caenorhabditis elegans* MTs are detected in the presynaptic region by electron tomography, although they run along the axis of the neurite and remain excluded from the SV pool and from dense projections of the active zone AZ (Stigloher et al., 2011). It follows that detection of MTs and other subcellular elements at presynaptic terminals depends on the type of synapse and the experimental method used in the analysis. An integrative representation of MT functions at a generic mammalian presynaptic terminal of the CNS is shown in **Figure 1**.

## Lessons From *Drosophila*

### Presynaptic NMJ Structure Depends on the MT Cytoskeleton

**FIGURE 2:**
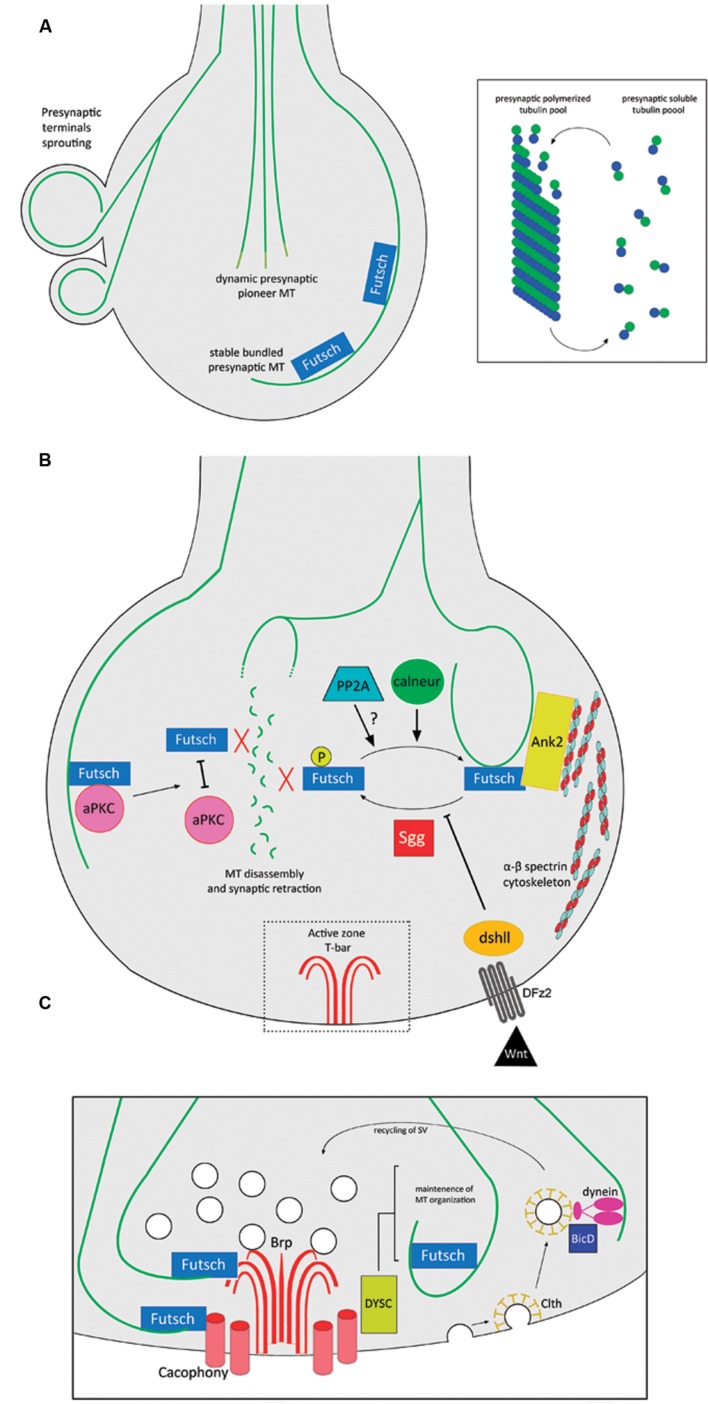
**MT functions and regulation at the presynaptic terminal of *Drosophila* NMJs.** **(A)** Different pools of MTs at presynaptic terminals of NMJs: Futsch-positive stable MTs and dynamic pioneer MT bundles. At presynaptic terminals, there is a balance between soluble and polymerized tubulin. **(B)** Futsch-dependent regulation of MT stability at presynaptic terminals. Un-phosphorylated Futsch interacts with MTs and promotes their stabilization. Sgg directly phosphorylates Futsch, detaching it from MTs, hence promoting MT disassembly and synaptic retraction, in a mechanism that is inhibited by the Wnt signaling pathway. The phosphatase calneur counteracts the effect of Sgg phosphorylation. It is also likely that the phosphatase PP2A participates in this mechanism. Ank2 links both MTs and Futsch to the spectrin cytoskeleton, conferring structural stability to the presynaptic terminal. Finally, disrupting the interaction between aPKC and Futsch detaches the latter from the MTs, inducing disassembly. **(C)** MT interactions with components of the NMJ active zone. Futsch links MTs with Brp and Cacophony at active zones. The active zone protein DYSC regulates the stability of the presynaptic MT cytoskeleton. The recycling of SV depends on the clth-mediated endocytosis of neurotransmitter-depleted SVs in a mechanism involving the MT cytoskeleton, dynein and BicD. Abbreviations: Ank2, Ankyrin 2; aPKC, atypical protein kinase C; BicD, Bicaudal-D; Brp, Bruchpilot; calneur, calcineurin; Clth, Clathrin; DFz2, *Drosophila* Frizzled-2; dshll, disheveled; DYSC, Dsychronic; PP2A, protein phosphatase 2A; Sgg, Shaggy.

One of the best-studied vertebrate synapse systems corresponds to the NMJ in the peripheral nervous system. MTs are present in NMJ presynaptic terminals (Fahim and Robbins, 1982; Sanes and Lichtman, 1999), where they play a key role in the maintenance of their structure. For example, in a mouse model for spinal muscular atrophy (SMA), the presynaptic MT cytoskeleton is dispersed and scattered (Torres-Benito et al., 2011). However, most of the knowledge of this particular synapse derives from studies using the *Drosophila* glutamatergic larval NMJ, where motor axons establish contact with muscle cells to form a branched synaptic terminal arbor with many varicosities or synaptic boutons (Ruiz-Cañada and Budnik, 2006). As synapses develop, emerging synaptic boutons are added between or following existing presynaptic boutons, in a mechanism that implies *de novo* generation or budding from existing terminals (Zito et al., 1999). A comprehensive review of the structure and development for this synapse can be found in Menon et al. (2013). Ultrastructure analysis determined the presence of MTs in presynaptic boutons of *Drosophila* NMJs (Atwood et al., 1993), where they organize as loops that form a thread-like structure through the presynaptic terminal and seem to play crucial roles in the establishment and maintenance of synapses (Roos et al., 2000; Pennetta et al., 2002). During the budding of synaptic boutons, presynaptic MT loops undergo a dynamic reorganization that involves MTs splaying apart into numerous fibers and then re-bundling again, after the new bouton starts to bud (Roos et al., 2000; Pennetta et al., 2002). Also, at *Drosophila* NMJ presynaptic terminals, an equilibrium exists between free subunits and polymers of tubulin (Yan and Broadie, 2007), and MTs protrude dynamically into the presynaptic terminals forming the so-called “pioneer presynaptic MTs” (Pawson et al., 2008) (**Figure 2A**). Presynaptic MTs interact directly with the *Drosophila* homolog of MAP1B/Futsch (Hummel et al., 2000), and such interactions promote MT stability at presynaptic boutons and synaptic growth (Roos et al., 2000; Godena et al., 2011). Futsch acts as a molecular link between presynaptic MTs and components of the active zone (Lepicard et al., 2014). Indeed, the atypical protein kinase C (aPKC) stabilizes the presynaptic MT cytoskeleton, promoting the interaction between Futsch and tubulin (Ruiz-Canada et al., 2004), and also presynaptic MT dynamics is regulated by post-translational modifications of Futsch. For instance, Futsch is phosphorylated by Shaggy (Sgg), the *Drosophila* homolog of glycogen synthase kinase-3β (GSK3β). Such phosphorylation induces Futsch detachment from MTs, which is ultimately linked to the disassembly of the presynaptic MT cytoskeleton (Franco, 2004; Gögel et al., 2006). This molecular pathway is proposed to be dependent on *wingless* (Wg), the *Drosophila* ortholog of the Wnt morphogen (see below). On the other hand, the protein phosphatase calcineurin is active at the resting calcium concentration in presynaptic terminals where it counteracts the effect of Sgg, decreasing the levels of P-Futsch, and therefore promoting presynaptic MT stabilization and synaptic growth (Wong et al., 2014). In addition, protein phosphatase 2A (PP2A) is also required for normal MT cytoskeletal organization, hence promoting presynaptic stability and synapse growth (Viquez, 2006).

It is important to mention that MTs are intertwined with other cytoskeletal elements to maintain presynaptic integrity. In particular, *Drosophila* Ankyrin 2 (Ank2) links the core presynaptic MT cytoskeleton with the membrane cytoskeleton enriched in spectrin scaffold proteins (Koch et al., 2008; Pielage et al., 2008), in a mechanism dependent on casein kinase 2 (CK2) functions (Bulat et al., 2014). Accordingly, disruption of the spectrin cytoskeleton produces synapse disassembly through disruption of presynaptic MTs (Pielage et al., 2005; Massaro et al., 2009). Two isoforms of the *Drosophila* giant Ankyrin 2 (Ank2-L and Ank2-XL) and Futsch form a membrane-associated MT organizing complex, where Ank2-L controls the synaptic localization of Any2-XL, and the latter synergistically with Futsch provides three-dimensional MT organization necessary for the establishment of appropriate presynaptic dimensions and release properties (Stephan et al., 2015). The stability of presynaptic MTs is also negatively regulated by the transcription factor Forkhead box class O (FoxO) since its overexpression drives NMJ overgrowth and MT destabilization. In addition, the fact that disruption of presynaptic MTs leads to a reduction of FoxO levels, argues that FoxO-dependent regulation is a component of the neuronal response to damage (Nechipurenko and Broihier, 2012). Finally, Sec8, a component of the exocyst is required for *in vivo* regulation of presynaptic MT formation and regulates synaptic growth and glutamate receptor trafficking (Liebl et al., 2005). A summary of the Futsch-dependent presynaptic MT regulation and its interaction with other cytoskeletal elements is represented in **Figure 2B**.

The evidence that proposes a structural role for presynaptic MTs is abundant; however, the MT tracks present at presynaptic terminals are also present in the proximity of active zones where they regulate neurotransmitter release and the endocytosis of SVs (**Figure 2C**). Futsch is found in an intermediate position between MTs and active zones, mediating the interaction with the active zone components *Bruchpilot* (Brp) and the calcium channel *Cacophony* (Lepicard et al., 2014). These data support the idea that Futsch locally stabilizes active zones by reinforcing their link with the underlying presynaptic MT cytoskeleton (Lepicard et al., 2014). The protein Bicaudal-D (BicD) plays roles in the transport of a subset of cargoes by the minus-end-directed MT motor dynein (Bianco et al., 2010). BicD binds directly with clathrin and interacts with components of the clathrin-mediated membrane trafficking pathway. In this context, BicD recognizes clathrin-coated SVs and mediates their recycling by facilitating dynein-based transport along presynaptic MT tracks (Li et al., 2010). Additionally, the scaffold protein Dsychronic (DYSC) is expressed presynaptically and is adjacent to the active zone. Loss of DYSC induces an unstructured Futsch-positive localization of MT within the presynaptic bouton (Jepson et al., 2014). In other synaptic structures, specifically at the *Drosophila* R8 photoreceptor, the redistribution of active zone components after extended exposure to light depends on MT cytoskeleton dynamics and on the kinesin, Imac (Sugie et al., 2015). Therefore, coordination between presynaptic MTs and active zone components plays a major role in regulating presynaptic physiology.

### *Trans*-synaptic Signaling Regulation of the Presynaptic MT Cytoskeleton

The development and maintenance of presynaptic terminal architecture and physiology is regulated by *trans*-synaptic signaling between the presynaptic neuron and the postsynaptic muscle cell in *Drosophila* NMJs. For example, pre- and postsynaptic *Drosophila* Teneurins (Ten-a and Ten-m, respectively) coordinately organize the presynaptic MT cytoskeleton and postsynaptic spectrin cytoskeleton (Mosca et al., 2012). However, major regulators of presynaptic functions are pathways involving retrograde signaling by two different morphogens: *Wingless* (Wg), the ortholog member of the vertebrate Wnt family, and *Glass bottom boat* (Gbb), the ortholog member of the bone morphogenetic protein (BMP) and transforming growth factor-β (TGFβ) family (Marqués, 2005; Collins and DiAntonio, 2007; Bayat et al., 2011). *Gbb*-null animals show reduced synapse area, alterations in presynaptic ultrastructure and several physiological defects at the NMJ (McCabe et al., 2003), a phenotype that is replicated in mutants for the *wishful thinking* (wit) gene that encodes for a BMP type II receptor (Aberle et al., 2002; Marqués et al., 2002). The same phenotype is also observed in mutants of the BMP type I receptors *Thickveins* (Tkv) and S*axophone* (Sax), and its downstream targets *Mothers against dpp* (Mad) and *Medea* (Med; Rawson et al., 2003; McCabe et al., 2004), providing strong support to the idea that the Gbb signaling pathway is a fundamental regulator of presynaptic structure at NMJs. As expected, mutants for Gbb, Tkv and Sax show a reduction and progressive loss of presynaptic Futsch-positive MTs (Ellis et al., 2010), while Wit mutants exhibit altered axonal transport that may reflect MT abnormalities (Aberle et al., 2002). Moreover, it has been described that strong synaptic connections at NMJs contain unbundled presynaptic MTs, suggesting that MT lattice organization could regulate the strength of neurotransmission. This process depends on the BMP signaling pathway since its disruption blocks synaptic strengthening (Ball et al., 2015). The protein Spartin inhibits BMP signaling, by promoting endocytic degradation of the presynaptic Wit receptor; thus Spartin loss-of-function increases levels of Futsch and stabilizes presynaptic MTs. Consistently, it has been proposed that the BMP pathway regulates synaptic growth in part by controlling presynaptic MT stability (Nahm et al., 2013). Similarly, *spichthyin*, the *Drosophila* ortholog of the proteins NIPA1 and ichthyin, antagonizes the stabilization of presynaptic MTs dependent on BMP signaling, since overexpression of *spichthyin* decreases Futsch levels at NMJs (Wang et al., 2007). The *Drosophila* ortholog of LIM-Kinase1 (DLIMK1) interacts with Wit and acts as a parallel BMP signaling pathway conferring synapse-stability, and it has been described that DLIMK1 filaments are closely associated with presynaptic MTs (Eaton and Davis, 2005). Finally, the Activin pathway, a member of the TGF-b superfamily of ligands, acts upstream of the Gbb pathway regulating synaptic growth at NMJs, and loss of Activin or its receptor Baboon (Babo) affects presynaptic MT stability, axonal transport and distribution of Futsch (Ellis et al., 2010).

In mammalian neurons, it has been observed that Wnt signaling participates in presynaptic terminal differentiation, enhancing the clustering of the presynaptic proteins Synapsin I and Bassoon (Hall et al., 2000; Varela-Nallar et al., 2009). *Wingless* (Wg) is expressed at presynaptic terminals, and its receptor DFrizzled2 (DFz2) is present in both the postsynaptic muscle and in the presynaptic motor neuron (Packard et al., 2002). When Wg levels are down-regulated, the emerging presynaptic boutons fail to develop active zones, and present an increased proportion of unbundled presynaptic MTs (Packard et al., 2002). In this context, Wg binds with the presynaptic DFz2/*arrow* receptor complex and activates the protein *disheveled*, blocking *Shaggy-*dependent phosphorylation of Futsch, hence promoting the organization of presynaptic MTs into bundles, budding of new boutons and synaptic growth (Miech et al., 2008). Interestingly, in mammals the homologous signaling pathway has been related with axonal elongation and MT dynamics at growth cones of mammalian neurons (Lucas et al., 1998; Goold et al., 1999), involving GSK3β phosphorylation upon MAP1B. Consistently, activation of the Wnt pathway inhibits such effects, resulting in increased MT stability (Salinas, 2007). The α subunit of the heterotrimeric protein Gα (Gαo) acts as a transducer of the Wg/DFz2 pathway, due to inhibition of Sgg phosphorylation upon Futsch; additionally, Gao promotes the interaction between Ank2 and MTs. These combined actions on MT binding proteins coordinately regulate the presynaptic MT cytoskeleton (Lüchtenborg et al., 2014). Finally, in the postsynaptic cell, DFz2 is endocytosed and imported into the nucleus in a mechanism dependent on the PDZ protein dGRIP, and alterations in this pathway interfere with the formation of synaptic boutons and lead to aberrant synaptic structures. However, dGRIP is also present at presynaptic terminals in the NMJ where it is enriched along the MT bundles that transverse the presynaptic terminal (Ataman et al., 2006). Such conspicuous localization may indicate that dGRIP regulates several steps of the presynaptic Wg signaling pathway that depend on the MT cytoskeleton.

### Presynaptic Microtubule-Related Neuronal Disorder Models in *Drosophila*

In recent years, *Drosophila* has been extensively used as a genetic model organism for the study of human diseases related with neurodegeneration, brain metabolism, and neuromuscular pathologies (Jeibmann and Paulus, 2009; Lloyd and Taylor, 2010). In the fruit fly, nearly 75% of all known human disease-related genes possess an ortholog (Bier, 2005). In this context, we will now refer to *Drosophila* disease models for human neuronal pathologies that underlie altered molecular mechanisms regulating the presynaptic MT cytoskeleton at NMJs.

### Hereditary Spastic Paraplegias

Hereditary spastic paraplegias (HSPs) are a group of neurodegenerative disorders characterized by the clinical feature of progressive lower limb spastic paralysis, due to a dysfunction of the corticospinal tract (Reid, 2003). These conditions are genetically heterogeneous since more than 20 loci have been related with the development of the pathologies (Reid, 2003). Amongst the proteins encoded by the HSP-related genes, there are different classes of molecules such as cell adhesion molecule L1 (L1-CAM; Jouet et al., 1994), the myelin proteins PLP and DM20 (Saugier-Veber et al., 1994) and the molecular motor KIF5A (Reid et al., 2002). However, about 40% of autosomal dominant HSP cases are related with different mutations in the SPG4 gene that encodes Spastin (Fonknechten et al., 2000; Meijer et al., 2002; Sauter et al., 2002). Human Spastin was first described as a member of the AAA (ATPases associated with diverse cellular activities) family of proteins (Hazan et al., 1999) that promotes MT disassembly *in vivo*, similar to its homologous MT-severing protein, katanin (Errico et al., 2002). The *Drosophila* ortholog of the human Spastin gene product (D-Spastin) is expressed during embryogenesis in both the central nervous system (CNS) and peripheral nervous system (PNS; Kammermeier et al., 2003). The fact that D-Spastin is functionally conserved with human Spastin allows the utilization of several *Drosophila* disease models for the study of the pathological conditions related with HSP (Roll-Mecak and Vale, 2005). In this context, D-Spastin is enriched in axons and presynaptic terminals of NMJs, where it regulates MT stability (Trotta et al., 2004). D-Spastin *knockdown* led to accumulation of stabilized presynaptic MTs that resulted in a reduction of the presynaptic terminal area and increased excitatory junctional current (EJC). Conversely, D-Spastin overexpression reduced the proportion of stable MTs that led to a decrease in the EJC amplitude (Trotta et al., 2004). However, it has also been communicated that D-Spastin-null animals showed a reduction in MTs within the presynaptic terminal of NMJs, suggesting that severing of MTs is required for the generation of short MT fragments that enter presynaptic terminals (Sherwood et al., 2004). This presynaptic phenotype produced by the loss-of-function of D-Spastin is recovered when the actin cytoskeleton-regulator protein p21-activated kinase 3 (pak3) is also down-regulated, since the aberrant diffuse MTs observed at distal tips of presynaptic terminals at NMJs of D-Spastin null *Drosophila* is recovered in animals that lack both pak3 and D-Spastin (Ozdowski et al., 2011), raising the possibility that combined regulation of actin and MT cytoskeleton dynamics could be concertedly controlled in the development of the HSP disease. It is important to mention that knockdown of *Drosophila* katanin, the homologous MT-severing protein of D-Spastin, also produces aberrant presynaptic phenotypes, indicating that appropriate regulation of MT dynamics seems to be crucial for the correct presynaptic terminal structure (Mao et al., 2014).

**FIGURE 3:**
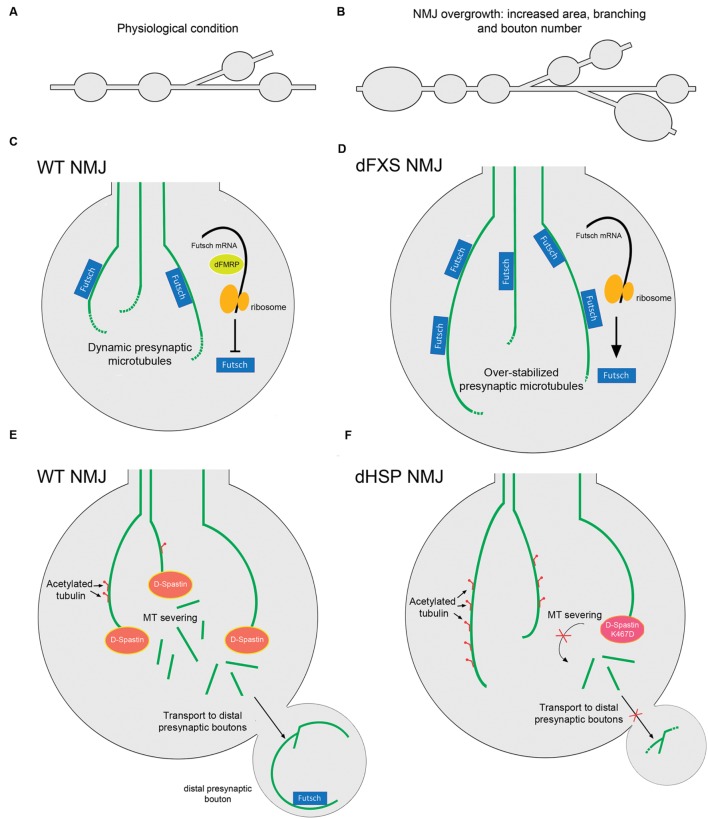
***Drosophila* models for dFXS and dHSP related with altered presynaptic MT structure.** Representation of the branched synaptic terminal arbor with varicosities or synaptic boutons, where motor axons establish contact with muscle cells in *Drosophila* larva, in **(A)** WT animals and in **(B)** dFXS model animals. In dFXS models, NMJ overgrowth is characterized by increased synaptic area, branching and presynaptic bouton number. **(C)** A WT NMJ presynaptic terminal. dFMRP negatively regulates Futsch translation, maintaining physiological levels of this protein and hence a dynamic pool of presynaptic MTs. **(D)** A dFXS NMJ presynaptic terminal. These mutants present abnormal NMJ overgrowth, as a consequence of the absence of dFMRP that leads to over-stabilized presynaptic MTs. **(E)** WT NMJ presynaptic terminals present physiological levels of stable MTs as D-Spastin severs MT bundles. Acetylated tubulin is represented as red motifs decorating MTs. Severed MTs are able to be transported to distal presynaptic terminals, where stable Futsch-positive MTs are found. **(F)** dHSP NMJ presynaptic terminals. Lack of D-Spastin leads to over stabilized MTs positive for acetylated tubulin. A similar phenomenon is observed when overexpressing the D-Spastin mutant K467D that lacks catalytic activity. Transport of MTs to distal presynaptic terminals is inhibited. Abbreviations: dFXS, *Drosophila* Fragile X Syndrome; dHSP, *Drosophila* Hereditary Spastic Paraplegias.

Patients with HSP present multiple different mutations in the loci encoding for Spastin, including point missense mutations and deletions. One of the best characterized mutations in humans is the substitution K388R that blocks ATP binding and hydrolysis, and that represents a pathogenic form of Spastin since it has been found in HSP patients (Fonknechten et al., 2000; Errico et al., 2002). Notably, when this substitution is generated in the fly sequence, the mutant D-Spastin is no longer able to disassemble MTs when overexpressed in *Drosophila* S2 cells (Roll-Mecak and Vale, 2005). Moreover, when expressing the D-Spastin K467R mutant (the corresponding mutation for K388R human Spastin) there is an increased proportion of stable MTs positive for acetylated α-tubulin in presynaptic terminals of NMJs, similar to the phenotype observed in D-Spastin knockdown assays (Orso et al., 2005). Remarkably, flies expressing K467R D-Spastin or the RNAi against D-Spastin that are fed with the MT-destabilizing drug vinblastine, reverse hyperstabilization of presynaptic MTs and any aberrant presynaptic phenotypes, raising the possibility that MT-targeting drugs could be used as an effective therapy in Spastin-associated HSPs (Orso et al., 2005). In addition, in humans there are two polymorphisms (S44L and P45Q) that are located outside the catalytic site of Spastin, and both L44 and Q45 are silent when expressed along with WT Spastin, but increase the severity of the pathology when expressed with the R388 mutation (McDermott et al., 2006). Similarly, the expression of human variants L44 and Q45 of Spastin in *Drosophila* presynaptic terminals exacerbate the effect of the R388 mutation (Du et al., 2010). Moreover, these transgenic flies therefore provides a model to analyze how human Spastin mutations regulates MT dynamics and presynaptic terminal stability (Du et al., 2010). In addition, a parallel therapeutic approach has been outlined using the *Drosophila* model. D-Spastin null *Drosophila* larvae grown at low environmental temperatures showed an evident rescue of their presynaptic morphology at NMJs, increased mobility and extended lifespan compared to those maintained at the control temperature, suggesting that mild hypothermia could be used as a neuroprotective technique in clinical treatment of HSP or other neurodegenerative diseases in humans (Baxter et al., 2014). Such observation may be linked to the fact that lower temperatures may mitigate mutant phenotypes, by slowing-down development and thus allowing compensational mechanisms. A representative diagram of the pathology related with D-Spastin and HSP is shown in **Figures 3A–F**.

Mammalian models for HSP show that Spastin deficiencies led to the generation of axonal swellings in neurons, which inhibit axonal MT-dependent transport of organelles such as mitochondria and APP-positive vesicles (Tarrade et al., 2006; Kasher et al., 2009; Denton et al., 2014). Nonetheless, a role for Spastin regulation of the MT cytoskeleton at presynaptic terminals has not yet been addressed in mammalian systems.

### *Drosophila* Fragile X Syndrome (dFXS)

Fragile X syndrome (FXS) is the most common form of inherited intellectual disability. It is caused by a CGG triplet expansion in the 5′-UTR of the *FMR1* gene that silences the expression of the FMRP protein. FMRP is an RNA-binding protein that selectively represses transcription of mRNAs that encode proteins involved in the regulation of dendritic spine morphology (Penagarikano et al., 2007; Bagni et al., 2012). The *Drosophila* ortholog of FMRP (dFMRP) has RNA-binding and protein–protein interaction characteristics similar to the human protein, and is preferentially expressed in the CNS during embryogenesis (Wan et al., 2000). In humans, there are two other proteins highly related to FMRP, termed FXR1 and FXR2 that may compensate FMRP deficiency (Myrick et al., 2015). In contrast, *Drosophila* only expresses dFMRP. The *Drosophila* fragile X syndrome (dFXS) model corresponds to dFMRP-null flies, which are adult viable and display normal development (Zhang et al., 2001). Interestingly dFMRP associates with Futsch mRNA, and negatively regulates its expression. Thus, it has been proposed that a major function of dFMRP is the negative regulation of Futsch in the nervous system, which in turn regulates MT-dependent presynaptic structure and function (Zhang et al., 2001). dFMRP functions also include morphological changes associated with neuronal plasticity, which are linked to Futsch decorated MTs at the presynaptic cytoskeleton (Gatto and Broadie, 2009). For example, induced neurodegeneration in hypomorphic Futsch flies, which is characterized by an abnormal MT network and defects in axonal transport, is suppressed when dFMRP is down-regulated (da Cruz et al., 2005). Accordingly, Futsch overexpression led to NMJ overgrowth, characterized by increased presynaptic area, branching and bouton number (Roos et al., 2000; Zhang et al., 2001), a phenotype that is recapitulated in dFMRP-null animals. Conversely, hypomorphic Futsch animals show reduced NMJ growth, similar to dFMRP gain-of-function mutants (Roos et al., 2000; Zhang et al., 2001). Additionally, an epistatic interaction of dFMRP acting upstream or in parallel with the MT-severing protein Spastin has been described, where dFMRP plays a crucial role in controlling presynaptic MT formation and axonal mitochondrial transport (Yao et al., 2011). Moreover, dFMRP down-regulates the levels and the spatial distribution of the Wg morphogen and participates in the translocation of the dFz2 receptor, opening the possibility that regulation of dFMRP over presynaptic MT stability could also be dependent on the Wg signaling pathway (Friedman et al., 2013). In **Figure 3B** the regulation of dFMRP over presynaptic MT dynamics at *Drosophila* NMJs is depicted. It is important to mention that the morphological changes affecting the structure of synapses at *Drosophila* NMJs are mirrored in mouse models of Fragile X syndrome. Mice FMRP interacts with MAP1B mRNA and represses its translation, and FMRP loss-of-function results in up-regulated MAP1B protein levels which leads to abnormally increased MT stability in neurons (Zalfa et al., 2003; Lu et al., 2004). MAP1B over-expression in cultured neurons resulted in decreased axonal elongation (Jimenez-Mateos et al., 2005). To date, there is a growing body of evidence supporting the idea of a presynaptic role for FMRP in mammalian neurons. FMRP and MAP1B mRNA were detected in axonal growth cones (Antar et al., 2006), and FMRP is located in presynaptic terminals and axons of different brain areas including the cortex and hippocampus, forming discrete granules called Fragile X granules (FXG; Christie et al., 2009). FMRP regulates calcium influx and neurotransmitter release at presynaptic terminals through interactions with different presynaptic proteins at both the CNS and PNS (Deng et al., 2013; Ferron et al., 2014) as well as the presynaptic capability of establishing synaptic contacts (Hanson and Madison, 2007). These results arise the possibility that FMRP could regulates presynaptic MT dynamic through MAP1B in mammalian neurons, and that this signaling pathway could be relevant in the physiopathology of Fragile X Syndrome.

### *Drosophila* Models for Other Pathologies

Mutations in the leucine-rich repeat kinase 2 (LRRK2) are linked to sporadic and familiar forms of Parkinson’s disease, and in *Drosophila*, mutated forms of its ortholog (dLRRK2) result in parkinsonism-like phenotypes (Liu et al., 2008). It has been described that at presynaptic terminals of NMJs, dLRRK2 phosphorylates Futsch and negatively regulates its interaction with MTs, leading to synaptic dysfunction due in part to altered MT dynamics. This phosphorylation is in a different residue from the one that Shaggy phosphorylates (Lee et al., 2010). This finding is in direct relation with the fact that the alteration of MT stability is an early event of dopaminergic neuron degeneration in mice, and that pharmacological stabilization of MTs may be used as a possible treatment against parkinsonism (Cartelli et al., 2013). Moreover, LRRK2 participates in canonical Wnt signaling as a scaffold, and it has been proposed that decreased LRRK2-mediated Wnt signaling underlies the neurodegeneration observed in Parkinson’s disease (Berwick and Harvey, 2012).

Trans-active response DNA binding protein (TDP-43) is a nuclear RNA binding protein that forms aggregates in about 95% of amyotrophic lateral sclerosis (ALS; Yang et al., 2014). TDP-43 associates with Futsch mRNA and regulates its expression at NMJs. Moreover, in an ALS model of induced TDP-43 aggregation, there is a significant reduction of Futsch mRNA at presynaptic terminals and a concomitant reduction of Futsch protein levels, which in turn result in disorganization of presynaptic MTs (Coyne et al., 2014). Interestingly, in spinal cords of ALS patients, MAP1B accumulates in motor neuron cell bodies, recapitulating abnormal Futsch in the *Drosophila* model for ALS (Coyne et al., 2014). Finally, missense mutations in the vesicle-associated membrane protein/synaptobrevin-associated membrane protein B (VAPB) gene are related with the development of ALS disease (Nishimura et al., 2004). In a *Drosophila* model for ALS, where the VAPB orthologous (dVAP33A) mutant protein aggregates, there is altered presynaptic BMP signaling that impairs presynaptic MT organization and Futsch immunostaining (Ratnaparkhi et al., 2008).

All the studies described before suggest that presynaptic MTs are a common target for several specific signaling pathways involved in the development of neurodegenerative diseases. This observation complements the notion that the disruption of MT-dependent axonal transport or aberrant MT dynamics contribute to the pathogenesis of multiple neurodegenerative diseases (Garcia and Cleveland, 2001; Chevalier-Larsen and Holzbaur, 2006). It follows that, to some extent, disruption of MT cytoskeleton at presynaptic terminals may contribute to pathophysiology of neurodegenerative diseases.

## Conclusion

Like the MT cytoskeleton in dendritic spines, the possibility that presynaptic MTs transiently protrude only in a small proportion of terminals in mammalian synapses needs to be explored using live cell imaging techniques. Recombinant fluorescent tagged-tubulin or plus tip MT-associated protein may be useful for such purposes, similar to the studies already performed at *Drosophila* NMJ presynaptic terminals, where tubulin-GFP transits between free subunits and polymers (Yan and Broadie, 2007) and EB1-GFP protrudes dynamically to presynaptic terminals leading to the so-called “pioneer presynaptic MT” (Pawson et al., 2008). Nonetheless, it has been previously reported that directly monitoring the dynamics of the MT cytoskeleton at presynaptic terminals of central synapses is difficult due to the small size of the terminals (Li and Murthy, 2001). If the presynaptic MTs correspond to specific subsets of cytoskeletal elements that are labile and unstable in the presence of calcium, their visualization is intrinsically difficult due to the calcium concentrations reached at presynaptic terminals, which are in the range of 5–200 μM evoking neurotransmitter release (Augustine, 2001). Such concentrations overlap with the calcium concentration of <100 μM that induces catastrophe MT events *in vitro* (O’Brien et al., 1997). The emerging idea that several neurodegenerative diseases are related to an altered presynaptic MT cytoskeleton structure has been extensively studied in *Drosophila* NMJs. Therefore, efforts from now on should focus on understanding the relevance of the presynaptic MT in mammalian models, in order to develop potential therapeutic approaches that target MT cytoskeleton dynamics. The extensive data discussed in this review lead to the conclusion that the concept of MTs being mere axonal transport tracks for SVs or other presynaptic organelles has to be revisited, considering that detection of MTs at presynaptic boutons may be conditioned by their intrinsic biochemical properties. Whether the MT cytoskeleton dynamically shapes presynaptic terminals and whether its presence is physiologically relevant will provide valuable new insights into the function of the cytoskeleton in these neuronal structures.

## Author Contributions

FJB and CG-B conceived and wrote the article.

## Conflict of Interest Statement

The authors declare that the research was conducted in the absence of any commercial or financial relationships that could be construed as a potential conflict of interest.
